# SparkGC: Spark based genome compression for large collections of genomes

**DOI:** 10.1186/s12859-022-04825-5

**Published:** 2022-07-25

**Authors:** Haichang Yao, Guangyong Hu, Shangdong Liu, Houzhi Fang, Yimu Ji

**Affiliations:** 1 School of Computer and Software, Nanjing Vocational University of Industry Technology, Nanjing, 210023 China; 2 School of Computer Science, Nanjing University of Posts and Telecommunications, Nanjing, 210023 China; 3 Jiangsu HPC and Intelligent Processing Engineer Research Center, Nanjing, 210003 China; 4 Institute of High Performance Computing and Bigdata, Nanjing University of Posts and Telecommunications, Nanjing, 210023 China

**Keywords:** Genome compression, Reference-based compression, Spark, Distributed parallel

## Abstract

Since the completion of the Human Genome Project at the turn of the century, there has been an unprecedented proliferation of sequencing data. One of the consequences is that it becomes extremely difficult to store, backup, and migrate enormous amount of genomic datasets, not to mention they continue to expand as the cost of sequencing decreases. Herein, a much more efficient and scalable program to perform genome compression is required urgently. In this manuscript, we propose a new Apache Spark based Genome Compression method called SparkGC that can run efficiently and cost-effectively on a scalable computational cluster to compress large collections of genomes. SparkGC uses Spark’s in-memory computation capabilities to reduce compression time by keeping data active in memory between the first-order and second-order compression. The evaluation shows that the compression ratio of SparkGC is better than the best state-of-the-art methods, at least better by 30%. The compression speed is also at least 3.8 times that of the best state-of-the-art methods on only one worker node and scales quite well with the number of nodes. SparkGC is of significant benefit to genomic data storage and transmission. The source code of SparkGC is publicly available at https://github.com/haichangyao/SparkGC.

**Supplementary Information:**

The online version contains supplementary material available at 10.1186/s12859-022-04825-5.

## Introduction

Genome plays an increasingly important role in human life, and genome technology has become a breakthrough in the treatment of new diseases [1]. Based on this, the European Molecular Biology Laboratory (EMBL), Gen-Bank of American National Center for Biotechnology Information (NCBI), and DNA Data Bank of Japan (DDBJ) are updating the database information every day. Because the cost of genome sequencing is continuously reducing, while the efficiency is increasing, the growth of biological data is amazing [2]. Such a huge amount of genomic data has posed great challenges to genomic data centers and genomic research institutions, such as in data storage, backup, migration, sharing, etc. [3]. The compression of genomic data naturally becomes the best choice to resolve the challenge. Although the general-purpose compression method can also be applied to genomic data, they do not use the characteristics of genomic data, thereby their compression ratio is limited. No matter what general-purpose compression method is used, the compression ratio can only reach 7:1 at most, which cannot completely resolve the challenge [4]. In recent years, researchers have proposed many special-purpose genome compression methods. Compared with the general-purpose compression methods, their compression ratio has been greatly improved [5].

As a data compression method, the compression ratio is the first evaluated factor in most situations, so this is the direction that genome compression researchers have been working on. But in the face of big data, the compression time is gradually emerging to be an urgent problem for researchers to resolve [6]. The compression method is always a trade-off between compression ratio and compression speed. The latest research results show that the compression ratio of pair-wise human genomes compressing has increased to an average of more than 300:1, but the compression time has also increased to more than 10 min per person [7]. The time cost may be tolerable when compressing a small amount of genomic data, such as 100 or 1 K human genomes. However, in the scenario of data archiving or data migration in the genomic data centers, it is very common to compress 10K, 100K, or even 1 million human genomes. At this time, the compression time required by current genome compression methods becomes intolerable.

Another problem is that with the continuous increase of genomic data, cloud storage, the storage mode specially designed for big data, has gradually entered the bioinformatics community [8]. Cloud storage enables users to use storage facilities on demand without the huge cost of building and maintaining expensive infrastructure. With recent services by Amazon and alike, it is possible to rent almost arbitrary configurations, which look logically as a single machine, but is in fact distributed. In this setup, when storing the genomic big data, the user does not need to care about using the cloud, since the infrastructure is hidden from the user. Although most genome compression methods can still be used in the cloud, they are almost single machine algorithms, which cannot fully utilize the computing power of distributed nodes. Developing a genome compression method that can be executed directly in distributed parallel systems has become a better solution [9].

Another advantage of studying parallel and scalable genome compression algorithms is that they can implement more complex compression schemes with high space-time requirements. The key factors of referential genome compression algorithms have been developed from maximum exact match (MEM) search to the prediction or calculation of precise differences between sequences. Solving the differences between two sequences is a global optimization problem, and solving the differences among large collections of sequences is an approximate NP-hard problem. They both have high computational complexity [6]. Distributed parallel algorithms can speed up these solvings that make the research of genome compression have more space.

In recent years, several distributed parallel frameworks have emerged to efficiently manage and process large datasets. The most popular of which are Hadoop [10] and Spark [11]. Both of them are open source big data frameworks of Apache. The core designs of the Hadoop framework are Hadoop Distributed File System (HDFS) and MapReduce. HDFS provides storage for massive data, while MapReduce provides the calculation for massive data. The MapReduce framework has a limitation on programmability though, as it requires the programmer to write code where the Map phase is always followed by the Reduce phase. Moreover, it saves intermediate data to the disk between Map phase and Reduce phase, which increases disk access overhead. Spark is a general parallel framework like MapReduce. It is open source by UC Berkeley AMP lab. Spark has the advantages of MapReduce and can also use HDFS as the distributed file system. But different from MapReduce, Spark allows programmers to perform many other transformations besides just Map and Reduce, while keeping data in memory between these transformations. These distributed parallel frameworks are different from the multi-core parallel schemes, which can be realized by modifying the code slightly. Only if the architectural details and the specific aspects of the considered framework are carefully taken into account for the algorithm design and implementation, genome compression can be developed well on these frameworks.

In this manuscript, we propose and implement a Spark based genome compression (SparkGC) method that allows running efficiently and cost-effectively on a scalable computational cluster to compress large collections of genomes. SparkGC utilizes Spark’s in-memory computation capabilities to improve the performance of genome compression. The contributions of this manuscript are summarized as follows:

- We proposed Spark based genome compression scheme for the first time. Although there are some genome compression methods based on distributed parallel computing, such as FastDRC [12], they are all based on MapReduce framework. To our best knowledge, there is no large collections of genomes compression method based on Spark. Our method proved the feasibility of compressing large collections of genomes via Spark. Furthermore, our research results indicated that Spark is more suitable for the iterative compression of large collections of genomes.
- We designed and implemented SparkGC: a production-quality and highly scalable large collections of genomes compression method. SparkGC meticulously designs the RDD (Resilient Distributed Datasets) transformations to keep data active in memory among the whole compression process. SparkGC improves the compression speed for about 4 times on the cluster with only one worker node and scales excellently by increasing the number of worker nodes.
- We optimized the framework by using Kyro serialization and broadcast variables compression that enable SparkGC to compress 1100 human genomes on a common computer with just 24 GB of RAM. We optimized the encoding scheme for the mapping results that makes SparkGC achieve the best compression ratio among all the state-of-the-art compression methods.

The remainder of this manuscript is organized as follows. In “Related works” section, we discuss the related works on genome compression and its parallelization. “Methodology” section presents the methodology of SparkGC. This is followed by “Results” section, which evaluates the performance of the proposed algorithms, including compression ratio, compression speed, scalability, robustness, and trade-off. We finally conclude the manuscript with “Conclusions” section.

## Related works

DNAZip [13] proposed in 2009 compressed James Watson’s 3 GB genome to 4 MB, so small, that it even can be sent by email attachment. The high compression ratio of the referential genome compression immediately aroused researchers’ interest, made more and more researchers focus on referential genome compression research and obtain many achievements. Table 1 summarises the related works of this paper. More works about genome compression can be referenced in review articles [14–16].

**Table 1:** Summary of the related works of this paper

Year	Name	Methodology	Characteristics	Parallelization
2009	DNAZip [13]	A serial of compression techniques (Variable integer (VINT), Delta positions (DELTA), SNP mapping (DBSNP), K-mer partitioning (KMER)) are taken together to reduce the size of a single genome	The SNP database dbSNP [24] and the mapping results between reference and target sequence have to be input as prerequisites, that limits its practicability	Serial
2012	BlockCompression [25]	The reference and target sequence are divided into fixed-length blocks. Matching are performed between the blocks	Compressed suffix tree is employed to save memory. Straightforward approximate matching is used to improve matching rate	Block-processing can be distributed on several CPUs
2013	FRESCO [17]	Suffix tree is used to index the reference sequence. The base after the exact match is saved as mutation	Three schemes (selecting a good reference, reference rewriting, and second-order compression) were proposed to improve the compression ratio	Serial
2015	COGI [18]	COGI transforms the genomic sequences to a bitmap, then applies a rectangular partition coding algorithm to compress the binary image	The reference sequence is selected using techniques based on co-occurrence entropy and multi-scale entropy. Compressing multiple sequences is supported by COGI, but the compression ratio decreases dramatically	Serial
2015	GDC2 [26]	GDC2 is developed to compress large collections of genomes. Second-order compression scheme and variable integer encoding scheme are employed to reduce the size of compressed files	GDC 2 is implemented in a multithreaded fashion. By default, GDC 2 uses 4 threads: 3 for the first level Ziv–Lempel factoring and 1 for the second-level factoring and arithmetic coding	Multithreaded parallel
2015	iDoComp [27]	Suffix array is used to index the reference sequence. Greedy matching scheme is used to match the reference and the target sequence	Suffix array has to be pre-computed and stored in the hard drive before compression	Serial
2016	NRGC [28]	NRGC uses the score based placement technique to quantify the differences between genome sequences, so as to obtain the best position of each target block on the reference blocks	NRGC has strict requirements on the similarity between the reference sequence and target sequence, which is prone to compression failure	Serial
2017	HiRGC [29]	In the pre-processing stage, HiRGC separates the target sequence file into the identifier, the length of each line, position intervals of lowercase letters and the letter ‘N’, special letters and base letters, and then different compression schemes are used to compress them according to their characteristics	The greedy matching scheme generates some suboptimal matching result	Serial
2018	SCCG [30]	SCCG optimized the greedy matching scheme of HiRGC. It combines the greedy matching with the segmentation matching used in NRGC, matches the target sequence to the corresponding reference segmentation first, improves the compression ratio	The compression time and memory consumption increase significantly	Serial
2019	HRCM [21]	HRCM supports both pair-wise sequence compression and multiple sequences compression. When multiple sequences are compressed, optimized second-order compression scheme is used to improve compression ratio	HRCM balances well the compression speed, compression ratio, and robustness, especially for large collections of genomes compression	Serial
2020	memRGC [7]	bfMEM algorithm [31] is used to save the compression time and memory usage. memRGC extends the MEMs if there are less than two SNPs between MEMs, that improves the compression ratio	INDEL (INsertion and DELetion) and more than two SNPs are omitted in the approximate matching of memRGC	multithreaded parallel
2021	HadoopHRCM [22]	HDFS and Map/Reduce architecture is employed to improve the compression speed of HRCM	Distributed parallel computing technology is introduced to the FASTA compression	Hadoop

In recent decade, the performance of referential genome compression method continues improving, including compression ratio, robustness, scalability and applicability. The object of compression is also extended from single sequence to large collections of sequences. Researchers improve the compression method from every stage of compression process, such as sequence pre-processing, reference selection, index building, matching scheme, parallel scheme, etc. Specifically, in terms of sequence pre-processing, earlier proposed methods, such as FRESCO [17] and COGI [18], converted all characters to lowercases or uppercases and treated all non-base characters as ‘n’. The pre-processing scheme reduces the matching complexity, but losses some information. Recent proposed methods basically preserved all information of target sequences, that is, achieved lossless compression. In terms of reference selection, COGI uses the technology based on co-occurrence and multi-scale entropy. FRESCO uses the technology based on RsbitX. RCC [19] and ECC [6] cluster the target genomes, and choose the centroid of each cluster as the reference sequence. In terms of index building, researchers employed different technologies (e.g. suffix tree, suffix array, hash array, compressed suffix tree, etc.) to adapt different scenarios. In terms of matching scheme, researchers proposed greedy matching, segmentation matching and approximate matching schemes. Due to the improving technologies and schemes, the compression ratio is getting better and better, from dozens [20] to more than 400:1 [6]. When the second-order compression scheme is employed, the compression ratio achieves even more than 2000:1 [21]. However, the cost is the increasing time complexity. With the exponential increase of genomic data, intolerable compression time emerges to be a problem that compression researchers have to work hard to resolve. Therefore, in order to reduce compression time, some researchers started to employ parallel technology. But the most used parallel technology is the straightforward multithreaded parallel technology. With the development of the big data processing technology, some Hadoop based genome compression method are proposed [22].But generally speaking, the research based on big data processing technologies still has a lot of work to be done. So far, to our best knowledge, there is no published research on Spark based genome compression, but only some Spark based genome analysis achievements [23].

## Methodology

This section will firstly introduce the architecture of SparkGC, and then describe separately how does SparkGC parallelize the compression tasks, and finally introduce the decompression.

### Architecture

**Fig. 1:**
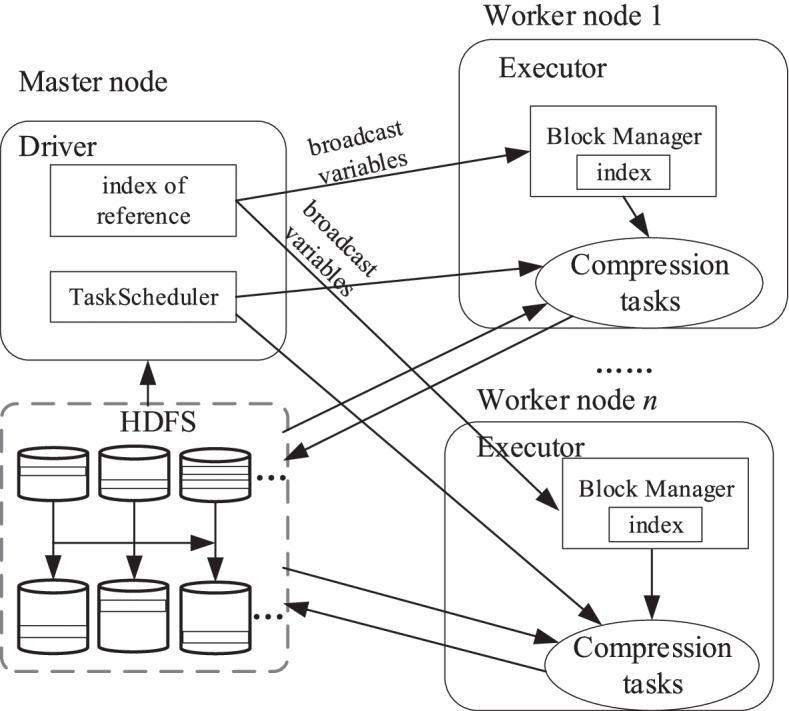
Architecture of Spark based genome compression

The architecture for large collections of genomes compression based on Spark is shown in Fig. 1. Spark architecture divides the computing cluster to master nodes and worker nodes. The compression algorithm is deployed on the master node, but the scheduling mechanism of Spark is migrating the computing tasks to nodes closest to the data, so the compression tasks will be scheduled to worker nodes. The *Driver* component of Spark executes the main function of genome compression in SparkGC. *TaskScheduler* component partitions the compression tasks and schedules them to each executor. Executor is a process running on worker node to execute compression tasks and cache intermediate results of RDD transformation. The master node reads the reference sequence from HDFS or local file system, and builds the index of the reference sequence. The driver broadcasts the reference sequence and its index as broadcast variables. The executor stores the broadcast variables to the *BlockManager* component. Broadcast variable is a shared variable mechanism of Spark. It enables the programs to send large size read-only data to all worker nodes. The worker nodes read the to-be-compressed sequences from HDFS before the compression and write the compressed results to HDFS after compression.

### Pre-processing

**Fig. 2:**
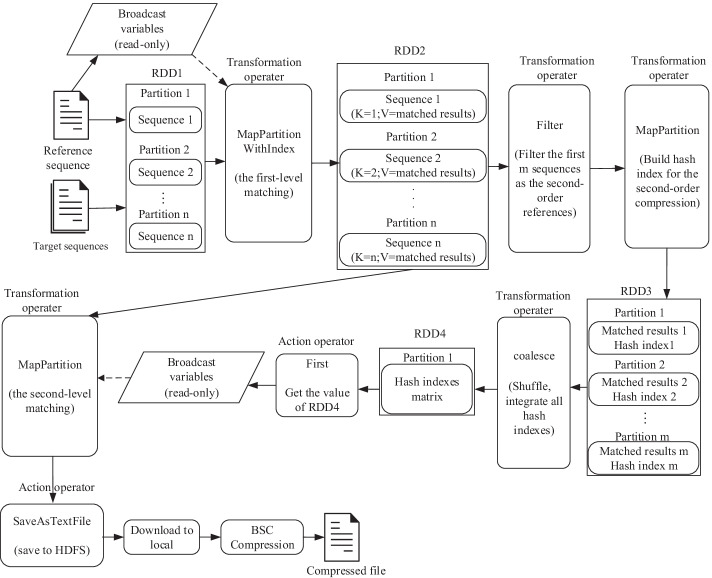
Data flow of SparkGC

The data flow of SparkGC is shown in Fig. 2. After each sequence file is read into memory, pre-processing of the sequence file follows closely. The sequence file is divided into two parts, base data, and auxiliary data. Base data refers to the base sequence composed of uppercase ACGT, and auxiliary data is the identifier, line break characters, special characters, and other information contained in the sequence. Because SparkGC is a lossless genome compression method, the auxiliary data of the to-be-compressed sequence cannot be lost. They are compressed independently with specific coding schemes at the pre-processing stage. The base data of the to-be-compressed sequence file saved as RDD to the memory of worker nodes for compression tasks. It is the first RDD of SparkGC data flow, so the RDD is indexed as RDD1 in this article. RDD is a logical entity of Spark. It is used as a whole, but actually the data of RDD are distributed in the memory of different worker nodes. The base data of one to-be-compressed sequence is one partition of RDD1. Each partition maps to a processing thread, which ensures that the process of these partitions is independent and concurrent.

The hash index built for the reference sequence data is on the master node. However, all worker nodes require reference sequence data and its hash index, so broadcast variables need to be created for them. In order to reduce the size of broadcast variables, Kryo [32] is used for serialization and compression of broadcast variables. The broadcast variables are set to read-only, which will not cause thread-safety problems. Then the broadcast variables are sent and cached in the memory of each worker node to prepare for the compression stage.

### Compression

The compression stage of SparkGC contains two steps which are referred to as the first-order compression and the second-order compression. The main task of the first-order compression is mapping the to-be-compressed sequences to the reference sequence based on the hash index, that generates the MEMs. The MEMs obtained at this stage are encoded as the tuple < position, length >. The mismatched sequence data is stored as the original characters. Therefore, after the first-level mapping, the original sequence data is converted to a new sequence composed of triple < position, length, mismatched string>. All the mapped results are not saved to the file system, but saved in the memory for the second-order compression. They are the second RDD of the data flow of SparkGC, i.e., RDD2. The mapped results of one to-be-compressed sequence are saved as one partition of RDD2. The partition is represented as < *key, value* >, where the *key* is the partition number and the *value* is the mapped results. The partition number is used to identify the sequence ID, so as to ensure the sequence order in subsequent processing. If the data amount of the *value* exceeds the memory limitation of the worker node, Kryo serialization will be used again to compress the data to prevent compression failure because of insufficient memory, that makes the compression of large collections of genomes successful on an ordinary computer.

After the first-order compression, a part of the compressed sequences will be regarded as the references of the second-order compression, hereinafter they are referred to as the second-order references. The second-order references are filtered out according to the sequence ID. Then, the hash index for the second-order references is built. The second-order references and their hash indexes are cached in memory as RDD3. The partitions of RDD3 are distributed on different worker nodes. Because like the first-order compression, all worker nodes need to use the second-order references and their hash indexes at the second-order compression stage. Therefore, all partitions in RDD3 are merged into one partition, i.e., RDD4. The master node then creates the broadcast variable based on RDD4 and sends it to all worker nodes. The merging will generate shuffle, that results in network transmission and disk access. However, after the first-order compression, the size of the compressed sequence has been reduced by more than 100 times compared to the original size, so the amount of shuffle data is not large. The first-order compressed results RDD2 is also kept in memory for the second-level matching.

The essence of the second-level matching is mapping the first-order compressed sequences to the second-order references by order using the hash indexes. The first-order compressed sequences are read from RDD2. The second-order references and their hash indexes are read from broadcast variables. After the second-level matching, the original first-level mapped results are converted to the second-level mapped results composed of triples < sequence ID, position, length> and triples < position, length, mismatched string>. Lastly, the second-order references are also compressed. The *i*-th second-order reference is mapped with the first to the (*i*-1)-th second-order references. In this way the second-order references can be losslessly reconstructed.

After the second-level mapping, all the mapped results are written to HDFS, one file for one sequence. These files will be downloaded to the local file system, compressed by BSC compression algorithm (http://libbsc.com/). So far, the whole compression is completed. SparkGC compression algorithm is summarized in Algorithm 1.
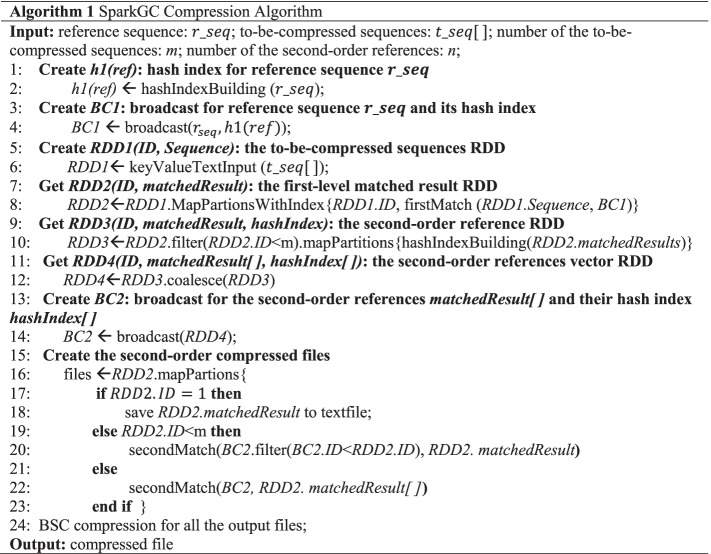


### Decompression

**Fig. 3:**
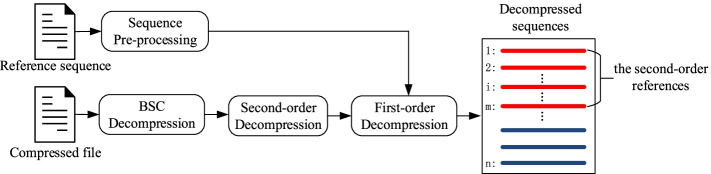
Overview of the decompression of SparkGC

Firstly, the compressed file is decompressed by BSC decompression algorithm to get all the second-order compressed data. The reference sequence is read and extracted in the same way as compression. But unlike compression, there is no need to build any hash index in decompression. So decompression and compression is asymmetric. Decompression requires much less memory and time. The target sequences are read by order, their base data and auxiliary data are reconstructed respectively. It is worth to note that SparkGC supports decompressing the sequences interested without decompressing all the sequences every time. The overview of the decompression is shown in Fig. 3. If the target sequence is one of the second-order references, the *i*-th target sequence only depends on its previous *i*-1 sequences. The target sequence can be totally decompressed without decompressing the remainder sequences. If the target sequence is not one of the second-order references, its decompression only depends on all the second-order references, other sequences don’t need to be decompressed. For large genomic datasets, this will save decompressors a lot of time.

Because more than 95% of decompression time is I/O time, the actual limitation of the decompression is the hard disk write speed. That is different from the compression which is CPU-bound and memory-bound. It has no effect to parallelize the decompression. Therefore, SparkGC does not implement the parallelization of decompression.

## Results

We evaluate the performance of SparkGC in this section. SparkGC was run on the cluster with 4 worker nodes and 1 master node. Each node is a common computer configured with 2 × 2.8 GHz Intel Xeon E5-2680 (20 cores) and 32 GB RAM. SparkGC was run over YARN (Yet Another Resource Negotiator) in the platform.

The datasets we selected firstly were 1000 Genome Project [33] which contains 1092 human genomes. In addition, we supplemented another 10 genomes HG13, HG16, HG17, HG18, HG19, HG38, K131 (the abbreviation of KOREF_20090131), K224 (the abbreviation of KOREF_20090224) [34], YH [35], and Huref [36]. These 10 human genomes are derived from different sequencing teams using different methods in different periods. They have different characteristics so that they are widely used in genome compression algorithms evaluation [7, 29, 30]. Therefore, our datasets totally contain 1102 human genomes and the total file size is about 3.11 TB. All the datasets can be downloaded from open access FTP server. Details of these datasets are provided in the Additional file 1.

### Compression ratio

**Fig. 4:**
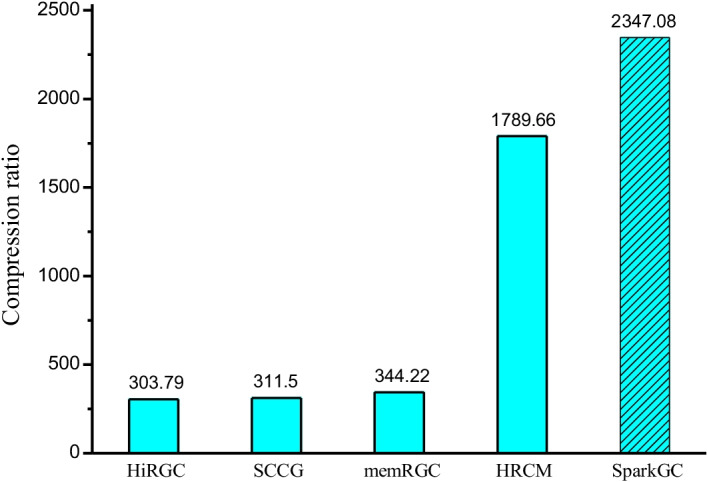
Compression ratio of SparkGC and the state-of-the-art methods

As a compression method, the compression ratio is always the first factor to be evaluated. Firstly we arbitrarily selected HG16 as the reference to compress other 1100 human genomes. In the robustness section, we evaluated the compression performance under different references. In addition, we also tested the compression ratio and compression time of the state-of-the-art genome compression methods in recent 4 years. They compressed the same 1100 human genomes under the same reference run on the same computers. These compression methods are HiRGC [29] proposed in 2017, SCCG [30] proposed in 2018, HRCM [21] proposed in 2019, and memRGC [7] proposed in 2020. Their compression ratios are shown in Fig. 4. Details of the experimental results are provided in Additional file 1: Table S3.

We can see from the Fig. 4, SparkGC achieved the best compression ratio among all the compression methods. The compression ratio of SparkGC is about 2347:1, it compressed the 3.11 TB original data to about 1387 MB. Compared to HiRGC, SCCG, and memRGC, the compression ratio is improved by 673%, 653%, and 582%. The compression ratio of SparkGC is greatly improved mainly because of the second-order compression scheme. After the first-level matching of each to-be-compressed sequence to the reference sequence, the matched results are not written to file, but saved in memory as intermediate data. Part of these intermediate sequences are selected as the second-order references to build hash index, then each first-order compressed sequence is compressed again according to the second-order hash index matrix. This compression scheme fully utilizes the similarity among the to-be-compressed sequences, greatly reduces the size of the compressed file. HRCM is also a second-order compression method, but compared to HRCM, the compression ratio of SparkGC is improved by 31%. The reason is that SparkGC uses BSC compression algorithm to compress the second-order compressed sequences.

### Compression speed

**Fig. 5:**
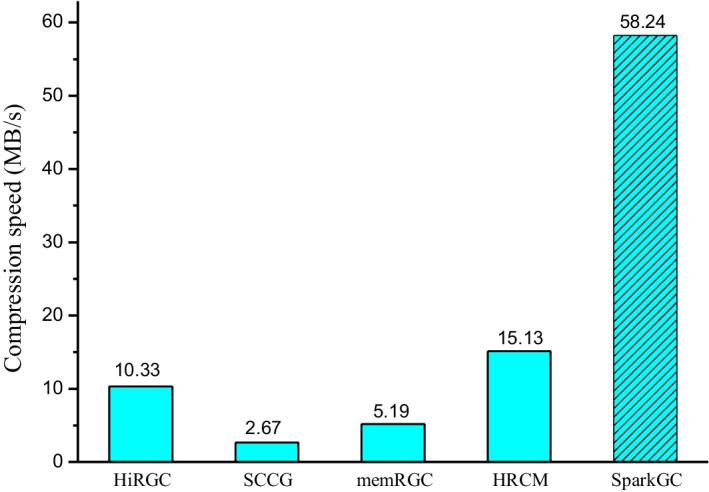
Compression speed of SparkGC and the state-of-the-art methods

The compression speed of compressing 1100 human genomes using HG16 as the reference sequence is shown in Fig. 5. Details of the experimental results are provided in Additional file 1: Table S4. Because SparkGC is a distributed parallel method, it will distribute the compression tasks on multiple nodes and run at the same time. In order to be as fair as possible, in this experiment, the number of worker nodes of SparkGC was set as 1, that is, SparkGC only used one worker node to perform the compression. The compression speed of multiple nodes will be illustrated in the scalability section. All compression time of this paper corresponds to the ‘real’ or wall-clock elapsed time. Each experiment was executed 3 times and the average time was recorded.

As can be seen from Fig. 5, although SparkGC only used one worker node to execute the compression, the compression speed achieved more than 58 MB/s, which is much higher than the best state-of-the-art methods. It only took 15.53 h to complete the compression of 3.11 TB genomic data. The compression speed is 5.63 times of HiRGC, 21.75 times of SCCG, 11.21 times of memRGC, and 3.85 times of HRCM. SCCG takes the most time, more than 14 days. It is hard to tolerate so much time to compress 1100 human genomes. SparkGC reduced the compression time of several days required by other methods to just more than half a day. The reason why SparkGC can achieve such high speed on one node is that the algorithm will make full use of the multi-thread of a single node automatically for compressing.

### Scalability

**Fig. 6:**
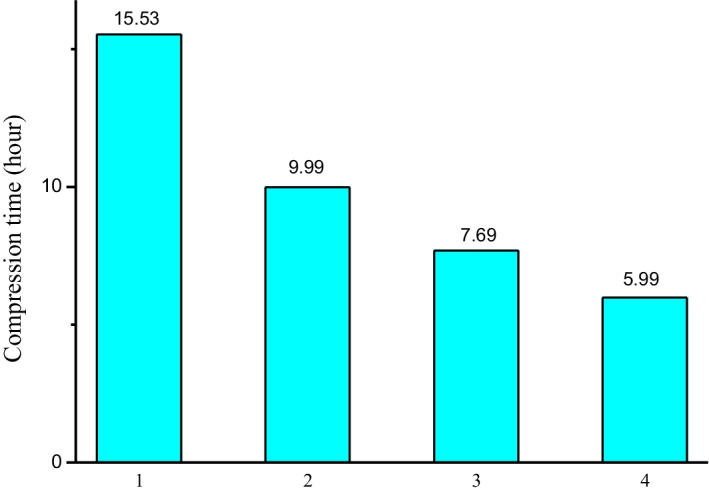
Total compression time under different number of worker nodes

The biggest advantage of SparkGC is not the performance on a single node, but its high scalability, which is the advantage that other methods do not have. We did a series of experiments to evaluate the scalability of SparkGC. Firstly we evaluated the compression speed of all chromosomes on the cluster with an increasing number of worker nodes activated, ranging from 1 to 4. The compression ratio of SparkGC does not correlate with the number of worker nodes, so the increasing number of worker nodes will not change the compression ratio. The total compression time of all chromosomes under different numbers of worker nodes is shown in Fig. 6. Here we observe that, with the increasing number of the worker nodes, the compression time decreases greatly. When the number of worker nodes is 4, SparkGC was able to compress the 3.11 TB genomic data to about 1387 MB in less than 6 h. The compression speed is about 151 MB/s.

In terms of runtime and parallelism, the following experiment evaluated the compression process of SparkGC from four stages:

1. Pre-processing stage completes the reading and hash index building of the reference sequence, and creating the broadcast variables.
2. First-order compression stage completes the first-level matching of all the to-be-compressed sequences to the reference sequence, then shuffles all the second-order references and their hash indexes, and creates the broadcast variables.
3. Second-order compression stage completes the second-level matching of all the first-order compressed sequences.
4. Post-processing stage downloads all the matched result files to the local file system and compresses them by BSC compression algorithm, then cleans up tasks on all worker nodes.

In these four stages, the first stage and the last stage cannot be parallelized, they are run only on the master node. Only the second stage and the third stage can be parallelized. Therefore, to evaluate the change of runtime of different stages with the increasing number of worker nodes, we illustrate the compression time of Chromosome 1 (abbreviate as Chr1) and Chromosome 13 (abbreviate as Chr13) in detail, as shown in Table 2.

**Table 2:** Runtime of different parts on different numbers of worker nodes

Chromosome	Stage	1 worker node		2 worker nodes		3 worker nodes		4 worker nodes	
		Time (s)	%	Time (s)	%	Time (s)	%	Time (s)	%
Chr1	Pre-processing	112	1.79	116	3.84	124	5.27	126	6.49
	First-order	5577	89.23	2618	86.72	2007	85.37	1648	84.81
	Second-order	531	8.50	254	8.41	188	8.00	136	7.00
	Post-processing	30	0.48	31	1.03	32	1.36	32	1.70
	Total	6250	100	3019	100	2351	100	1943	100
Chr13	Pre-processing	62	3.58	70	6.42	70	9.06	70	10.74
	First-order	1520	87.76	921	84.50	625	80.85	512	78.53
	Second-order	120	6.93	69	6.33	47	6.08	39	5.98
	Post-processing	30	1.73	30	2.75	31	4.01	31	4.75
	Total	1732	100	1090	100	773	100	652	100

It can be seen from Table 2 that the pre-processing stage and the post-processing stage did not save time with the increase of worker nodes, on the contrary, their runtime increased with the increase of worker nodes. Because with the increase of worker nodes, the program needs to initialize all worker nodes, and broadcast variables need to be sent to all worker nodes, which increases the runtime. Similarly, at the post-processing stage, the increase of worker nodes will lead to a longer clean-up time. Observing the first-order compression stage and the second-order compression stage will find that these two stages scaled quite well. For example, to Chr1, when the number of worker nodes was 1, 2, and 4, the average first-order compression time of each chromosome was 5, 2.4, and 1.5 s respectively, and the average second-order compression time of each chromosome was 0.48, 0.23, and 0.12 s respectively. To Chr13, it was 1.38, 0.83, and 0.46 s respectively at the first-order compression stage and 0.11, 0.06, and 0.03 s respectively at the second-order compression stage. The runtime was almost decreasing at the linear speed. From the percentage of runtime at each stage, the percentage of serial computing was gradually increasing, while the percentage of parallel computing was gradually decreasing, so the overall runtime showed a sublinear downward trend.

**Fig.7:**
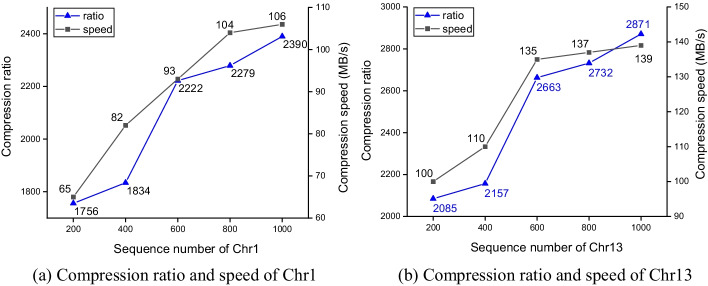
Compression performance to the different number of target sequences

The above experiments are all compressing 1100 human genomes. We are very interested in how the compression ratio and compression speed of SparkGC scale with the number of the to-be-compressed sequences. Because in the actual compression scenario, SparkGC will compress any size of genomic data sets. We evaluated the compression ratio and compression speed of Chr1 and Chr13 when the sequence number was 200, 400, 600, 800, and 1000 respectively, as shown in Fig. 7. In this experiment, the number of worker nodes was 3.

We can see from Fig. 7 that SparkGC also scaled quite well to the number of the to-be-compressed sequences. The compression ratio and compression speed of Chr1 and Chr13 both increased with the increase of the to-be-compressed sequences. The compression ratio of Chr1 gradually increased from 1756:1 to 2390:1, and that of Chr13 increased from 2085:1 to 2871:1. The compression speed of Chr1 increased gradually from 65 MB/s to 106 MB/s, and that of Chr13 increased from 100 MB/s to 139 MB/s. The compression ratio of SparkGC increases with the increase of the to-be-compressed sequences is because in that case, the percentage of the second-order references decreases. The default number of the second-order references of SparkGC is 40. The compression ratio of the second-order references is low, because the *i*-th second-order reference is compressed only using the first to the (*i*-1)-th sequences as references. So the smaller *i* is, the smaller the compression ratio is. The compression speed of SparkGC also increases with the increase of the to-be-compressed sequences, because the percentage of the first-order compression time and the second-order compression time increases. The compression speed and compression ratio of Chr13 is higher than Chr1 is because of the different similarity of different chromosomes. Generally, the greater the similarity of chromosomes, the higher the compression ratio and the faster the compression speed [37].

### Robustness

The compression ratio of the referential compression method is easily affected by the reference sequence. Therefore, some researchers studied the reference selection [6] [17] [19]. However, with the increase of the to-be-compressed sequences, the selection of reference sequences becomes hard due to its high time complexity. We evaluated the affection of different references to the performance of SparkGC by selecting 6 genomes with different characteristics to compress 1100 human genomes. The compressed size and compression time of Chr1 and Chr13 under different references are shown in Tables 3 and 4 respectively. In the two tables, AVG represents the average value under different references, it is computed by (1).

1 $$AVG = {{\sum\nolimits_{i = 1}^{n} {s_{i} } } \mathord{\left/ {\vphantom {{\sum\nolimits_{i = 1}^{n} {s_{i} } } n}} \right. \kern-\nulldelimiterspace} n}$$

where $s_{i}$ represents the compressed file size, and the *n* value is the number of references. SD is the standard deviation of all values, represents the degree of dispersion. It is computed by (2).

2 $$SD = \sqrt {\frac{1}{n}\sum\nolimits_{i = 1}^{n} {\left( {x_{i} - \overline{x} } \right)^{2} } }$$

where $\overline{x}$ represents the AVG value of the case. It can be seen from Table 3 that the compression ratio of SparkGC achieved the best results under all reference sequences, and the influence of reference on SparkGC was very small. The difference betweeen the maximum and minimum compressed size of Chr1 is 15 MB, and of Chr13 is 8 MB. Compared with the original data of 264,994 MB and 122,492 MB respectively, these differences can be ignored. The compressed results of HiRGC, SCCG, and memRGC under different reference sequences are quite different, especially to Chr1. The maximum compressed size of HiRGC, SCCG, and memRGC of Chr1 is 5.15 times, 5.24 times, and 5.75 times that of the minimum compressed size, respectively. When HG13 was the reference sequence, SCCG even failed to compress all sequences. The reason why SparkGC is less affected by the reference sequence is that if the similarity between the reference sequence and the to-be-compressed sequence is low, many identical mismatched fragments will be generated after the first-level matching, and these mismatched fragments will be matched and compressed in the second-level matching, so the compression result has little relationship with the similarity between the reference sequence and the to-be-compressed sequence. HRCM is also one of the second-order compression methods, so HRCM also performs well in robustness.

**Table 3:** Compressed size under different references

Chromosome	Original size (MB)	Method	Compressed size (MB) under different references						AVG	SD
			HG13	HG16	K131	YH	Huref	HG00096		
Chr1	264,994	HiRGC	2474	1313	828	750	1026	480	1145	647
		SCCG	2430	1284	775	706	986	464	1107	643
		memRGC	2324	1192	650	593	887	406	1009	638
		HRCM	126	140	156	154	151	136	144	11
		SparkGC	**123**	**115**	**130**	**128**	**125**	**115**	**123**	**6**
Chr13	122,492	HiRGC	333	296	413	394	314	219	328	64
		SCCG	/	289	390	375	308	219	/	/
		memRGC	288	254	333	319	262	190	274	47
		HRCM	48	56	66	65	63	57	59	6
		SparkGC	**44**	**44**	**52**	**52**	**50**	**45**	**48**	**3**

‘/’ indicates the method fails to compress the chromosome. Bold indicates the best value of the case

**Table 4:** Compression time under different references

Chromosome	Method	Compression time (hour) under different references						AVG	SD
		HG13	HG16	K131	YH	Huref	HG00096		
Chr1	HiRGC	11.95	7.40	8.75	8.82	10.01	6.55	8.91	1.75
	SCCG	21.25	21.91	37.73	37.11	39.86	36.66	32.42	7.73
	memRGC	20.49	12.05	16.09	18.45	16.53	11.07	15.78	3.32
	HRCM	11.19	8.28	9.60	8.40	9.76	5.72	8.82	1.69
	SparkGC	**0.70**	**0.54**	**0.57**	**0.49**	**0.63**	**0.40**	**0.56**	**0.10**
Chr13	HiRGC	2.67	2.43	2.37	2.45	2.48	2.41	2.47	0.10
	SCCG	/	24.15	18.61	23.44	12.37	10.17	/	/
	memRGC	9.07	8.13	14.62	9.82	11.25	8.23	10.19	2.25
	HRCM	1.64	1.43	1.47	1.40	1.44	1.25	1.44	0.11
	SparkGC	**0.2**	**0.18**	**0.19**	**0.17**	**0.16**	**0.15**	**0.18**	**0.01**

‘/’ indicates the method fails to compress the chromosome. Bold indicates the best value of the case

As can be seen from Table 4, SparkGC performed better in the robustness of compression time. The SD values of Chr1 and Chr13 are only 0.1 and 0.01 respectively, which are far lower than other compression methods. In terms of compression time, the maximum and smallest minimum compression time of Chr1 are 42 min and 24 min respectively; the maximum and minimum compression time of Chr13 are 20 min and 9 min respectively, and the difference is very small.

### Discussion

**Fig. 8:**
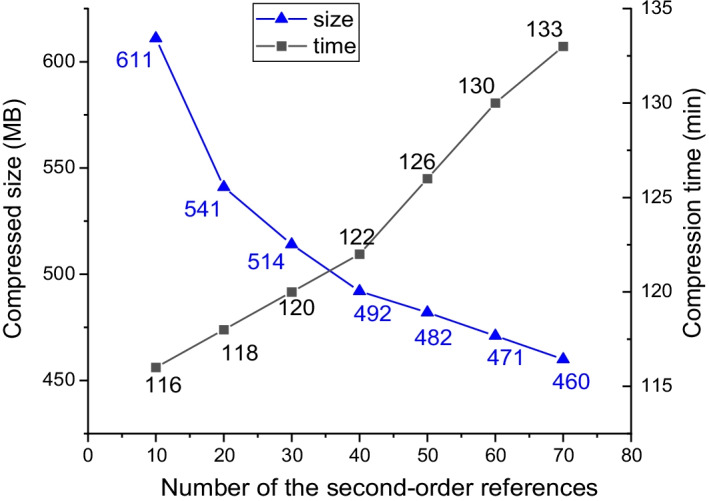
Trade-off between compression ratio and compression speed

Data compression is always a trade-off between compression ratio and compression speed, so is SparkGC. When the reference sequence is determined, the most important factor affecting compression ratio and compression speed is the number of the second-order references. In the second-level matching, the more the second-order references are, the greater the probability of matching successfully, so the compression ratio is higher. However, the cost is that the shuffle time and the hash index building time of the second-order references, the transferring time of broadcast variables, and the search time in hash index will be longer. We evaluated the trade-off between compression ratio and compression speed of SparkGC under 7 different numbers of the second-order references, as shown in Fig. 8. In this experiment, we evenly selected 8 chromosomes of our datasets for compressing. They are Chr1, Chr4, Chr7, Chr10, Chr13, Chr16, Chr19, and Chr22. The total file disk size of these chromosomes is about 1 TB.

We can see from the figure, with the increase of the number of second-order references, the compressed size decreases, and the compression time increases. The compressed size decreases from 611 MB when the number of second-order references is 10 to 460 MB when the number of second-order references is 70, the reduction rate is 24.7%. However, the compression time increases from 116 to 133 min, with an increase of 14.7%. Therefore, the compressors can choose the appropriate number of the second-order references according to their own needs.

**Fig. 9:**
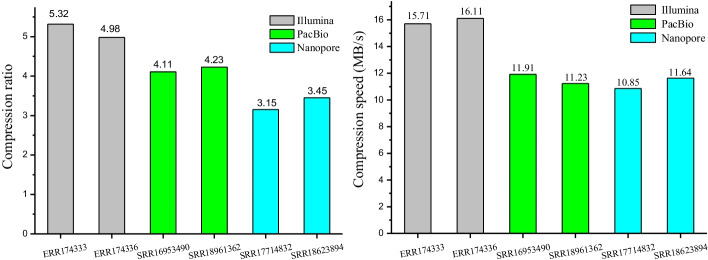
Compression ratio and speed on FASTQ data sets

In order to expand the applicability of the method, we developed sub-modules to compress FASTQ sequence based on the proposed methodology. Furthermore, we used data sets generated by different sequencing technologies including new and traditional ones to evaluate the FASTQ compression modules. The sequencing technologies we selected are Illumina, PacBio, and Oxford Nanopore. Details of the data sets are provided in Additional file 1: Table S2. The compression ratio and speed are shown in Fig. 9.

From Fig. 9, it is shown that SparkGC successfully work on all test data sets. However, the performance changes with different sequencing technologies. It obtains superior performance on the second generation sequencing platform Illumina to the third generation sequencing platforms PacBio and Oxford Nanopore. The reason is that the third generation sequencing technologies obtain longer read length, but which is accompanied by a relatively higher error rate. The error bases result in more fragments when matching the reference sequence, which affects the compression performance. In addition, the unfixed read length value also consumes a certain amount of storage space.

The evaluation of a genome compression method must take into account the main memory usage. Compared with the stand-alone programs, it is more complex to discuss the memory usage of SparkGC. Because the tasks of the master node and each worker node are different, the memory usage is different. The master node is responsible for reading reference sequences, the aggregation of the first-order compression results, and the hash index building. The memory footprint of the master node is affected by the size of the reference sequence and the number of second-order references. The worker node is responsible for reading the to-be-compressed sequences, the first-order compression, and the second-order compression. The memory usage is related to the number of to-be-compressed sequences. From our experimental observation, compressing 1100 human genomes consumes the most memory. However, whether on the master node or worker node, the memory footprint of SparkGC is less than 20 GB.

## Conclusions

This research proposes and implements a genome compression method based on Apache Spark. It can run efficiently on a multi-node cluster to compress large collections of genomes. Compared to the state-of-the-art genome compression methods, the compression ratio and speed are both recognizably improved. Besides, the method has good scalability and robustness. It will greatly benefit the storage of large genomic datasets. However, it should be noted that developing Spark based programs is not a trivial task. As such, they have largely only been embraced in the technology sector. Making Spark based genome compression method easy to use and extend for more non-computer science professionals is our goal at the next stage.

## Supplementary Information

**Additional file 1**. Details of the data sets and experimental results.

## Data Availability

The datasets analysed during the current study are all available in the public server and can be downloaded freely. Details of these datasets are provided in the Additional file 1. The source code of the current study is available at https://github.com/haichangyao/SparkGC.
