# Supplementary material for: SparkGC: Spark based genome compression for large collections of genomes
Source: BMC Bioinformatics. 2022 Jul 25;23:297. doi: 10.1186/s12859-022-04825-5 (PMC9310413; doi:10.1186/s12859-022-04825-5)
Supplement: Supplementary file 1 — Additional file 1. Details of the data sets and experimental results. [file 12859_2022_4825_MOESM1_ESM.docx]

# Additional file 1

This document provides the details of the data sets and experimental results.

# 1 Data sets

## 1.1 Details of the benchmark data sets used in the experiments

**Table S1.** Details of the benchmark data sets used in the experiments

| **Species** | **Data sets** | **Number of Chromosomes** | **Size** | **Retrieved from** |
| --- | --- | --- | --- | --- |
| Homo sapiens | HG13 | 24 | 2967.55 | ftp://hgdownload.soe.ucsc.edu/goldenPath/hg13/chromosomes/ |
|  | HG 16 | 24 | 2986.53 | ftp://hgdownload.soe.ucsc.edu/goldenPath/hg16/chromosomes/ |
|  | HG 17 | 24 | 2992.98 | ftp://hgdownload.soe.ucsc.edu/goldenPath/hg17/chromosomes/ |
|  | HG 18 | 24 | 2996.52 | ftp://hgdownload.soe.ucsc.edu/goldenPath/hg18/chromosomes/ |
|  | HG 19 | 24 | 3011.38 | ftp://hgdownload.soe.ucsc.edu/goldenPath/hg19/chromosomes/ |
|  | HG 38 | 24 | 2996.52 | ftp://hgdownload.soe.ucsc.edu/goldenPath/hg38/chromosomes/ |
|  | K131 | 24 | 2986.77 | ftp://ftp.kobic.re.kr/pub/KOBIC-KoreanGenome/  KOREF_20090131/fasta/ |
|  | K224 | 24 | 2986.75 | ftp://ftp.kobic.re.kr/pub/KOBIC-KoreanGenome/  KOREF_20090224/fasta/ |
|  | YH | 24 | 2986.75 | ftp://public.genomics.org.cn/BGI/yanhuang/fa/ |
|  | HuRef | 24 | 2993.95 | https://www.ncbi.nlm.nih.gov/nuccore Accession:CM000462-CM000485 |

## 1.2 The 1000 Genomes Project data sets

The 1000 Genomes Project data sets were retrieved based on the VCF files downloaded from 1000GP FTP server and the script vcf2fasta provided by GDC2 [1].

The reference sequences of assembled chromosomes can be downloaded from:

ftp://ftp.ncbi.nlm.nih.gov/genomes/H_sapiens/Assembled_chromosomes/seq/

The VCF files can be downloaded from:

ftp://ftp.ncbi.nlm.nih.gov/1000genomes/ftp/phase1/analysis_results/integrated_call_sets/

The details of how to use the reference sequences and the VCF files to retrieve the FASTA files are shown in the supplementary material of GDC2.

## 1.3 Details of FASTQ data sets

**Table S2.** Details of FASTQ data sets used in the experiments

| **ID** | **Platforms** | **Species** | **Size (MB)** |
| --- | --- | --- | --- |
| ERR174333 | Illumina HiSeq 2000 | Homo sapiens | 48692 |
| ERR174336 | Illumina HiSeq 2000 | Homo sapiens | 49610 |
| SRR16953490 | PACBIO_SMRT (PacBio RS II) | Homo sapiens | 875 |
| SRR18961362 | PACBIO_SMRT (Sequel) | Homo sapiens | 446 |
| SRR17714832 | OXFORD_NANOPORE（GridION） | Homo sapiens | 140 |
| SRR18623894 | OXFORD NANOPORE（MinION） | Homo sapiens | 1024 |

# 2 Additional experimental results

**Table S3.** Compressed size of SparkGC and the state-of-the-art methods

| **Chromosome** | **Original**  **file size (MB)** | **Compressed size (MB) by** | | | | |
| --- | --- | --- | --- | --- | --- | --- |
|  |  | **HiRGC** | **SCCG** | **memRGC** | **HRCM** | **SparkGC** |
| Chr1 | 264,994 | 1,312.68 | 1,284.34 | 1,192.36 | 139.82 | **115.18** |
| Chr2 | 258,659 | 1,029.51 | 1,007.72 | 936.45 | 144.60 | **120.06** |
| Chr3 | 210,603 | 517.09 | 502.23 | 444.50 | 118.18 | **96.52** |
| Chr4 | 203,098 | 647.97 | 629.50 | 567.30 | 121.29 | **98.58** |
| Chr5 | 192,416 | 404.91 | 390.63 | 342.44 | 146.22 | **86.34** |
| Chr6 | 181,936 | 488.95 | 472.88 | 420.69 | 151.21 | **89.09** |
| Chr7 | 169,217 | 596.40 | 582.19 | 523.14 | 100.99 | **81.26** |
| Chr8 | 155,608 | 433.54 | 421.88 | 374.11 | 92.01 | **73.40** |
| Chr9 | 150,130 | 729.60 | 712.67 | 643.92 | 73.40 | **58.16** |
| Chr10 | 144,163 | 442.64 | 431.53 | 385.45 | 82.72 | **65.58** |
| Chr11 | 143,548 | 383.62 | 372.35 | 324.88 | 79.01 | **63.57** |
| Chr12 | 142,270 | 457.30 | 445.55 | 399.72 | 80.36 | **63.29** |
| Chr13 | 122,492 | 296.18 | 288.70 | 254.48 | 56.20 | **44.04** |
| Chr14 | 114,124 | 461.89 | 452.26 | 419.95 | 53.10 | **40.87** |
| Chr15 | 109,050 | 235.42 | 228.64 | 201.33 | 48.59 | **37.69** |
| Chr16 | 96,103 | 276.43 | 268.72 | 236.86 | 53.04 | **41.02** |
| Chr17 | 86,315 | 247.27 | 239.97 | 212.18 | 45.95 | **35.77** |
| Chr18 | 83,045 | 181.86 | 182.25 | 153.21 | 44.28 | **33.79** |
| Chr19 | 62,845 | 148.36 | 141.22 | 116.86 | 38.49 | **29.44** |
| Chr20 | 67,021 | 164.54 | 160.20 | 143.18 | 35.95 | **27.02** |
| Chr21 | 51,166 | 372.86 | 367.39 | 350.65 | 23.69 | **17.27** |
| Chr22 | 54,495 | 220.91 | 216.80 | 199.51 | 23.91 | **17.70** |
| ChrX | 163,606 | 594.26 | 580.25 | 546.56 | 64.86 | **14.99** |
| ChrY | 29,084 | 73.80 | 72.59 | 69.42 | 1.63 | **1.48** |
| Total | 3,255,987 | 10,717.98 | 10,452.46 | 9,459.14 | 1,819.33 | **1,387.25** |

*Note: Bold indicates the best value of this case.*

**Table S4.** Compression time of SparkGC and the state-of-the-art methods

| **Chromosome** | **Compression time ( hour ) by** | | | | |
| --- | --- | --- | --- | --- | --- |
|  | **HiRGC** | **SCCG** | **memRGC** | **HRCM** | **SparkGC** |
| Chr1 | 7.40 | 21.91 | 12.05 | 8.28 | **1.74** |
| Chr2 | 6.62 | 22.47 | 10.97 | 6.42 | **1.11** |
| Chr3 | 5.94 | 19.11 | 8.73 | 4.11 | **0.84** |
| Chr4 | 4.98 | 17.54 | 7.99 | 3.24 | **0.99** |
| Chr5 | 4.82 | 19.00 | 7.53 | 3.64 | **0.91** |
| Chr6 | 4.94 | 16.39 | 7.23 | 3.46 | **0.86** |
| Chr7 | 4.24 | 15.08 | 6.74 | 3.47 | **0.97** |
| Chr8 | 3.90 | 14.32 | 6.05 | 2.60 | **0.51** |
| Chr9 | 3.67 | 12.66 | 11.82 | 2.51 | **0.68** |
| Chr10 | 3.28 | 12.43 | 5.30 | 2.46 | **0.66** |
| Chr11 | 5.68 | 25.01 | 6.60 | 2.22 | **0.85** |
| Chr12 | 3.90 | 11.95 | 9.71 | 2.97 | **0.67** |
| Chr13 | 2.43 | 24.15 | 8.13 | 1.43 | **0.48** |
| Chr14 | 2.52 | 11.23 | 11.92 | 1.61 | **0.53** |
| Chr15 | 2.14 | 14.84 | 11.39 | 1.43 | **0.58** |
| Chr16 | 2.39 | 9.01 | 5.51 | 1.34 | **0.43** |
| Chr17 | 2.48 | 8.28 | 5.26 | 1.73 | **0.28** |
| Chr18 | 1.41 | 15.23 | 5.32 | 0.92 | **0.37** |
| Chr19 | 1.92 | 6.62 | 3.66 | 1.22 | **0.33** |
| Chr20 | 1.68 | 6.85 | 3.83 | 0.84 | **0.33** |
| Chr21 | 3.18 | 4.91 | 6.20 | 0.56 | **0.26** |
| Chr22 | 1.22 | 6.71 | 3.59 | 0.56 | **0.23** |
| ChrX | 6.19 | 20.56 | 7.64 | 2.53 | **0.82** |
| ChrY | 0.62 | 1.87 | 1.13 | 0.24 | **0.10** |
| Total | 87.54 | 338.12 | 174.29 | 59.78 | **15.53** |

*Note: Bold indicates the best value of this case.*

# 3 References

1. Deorowicz S, Danek A, Niemiec M. GDC 2: Compression of large collections of genomes. Scientific Report. 2015; 5:1-12.
